# Rhizobia and their bio-partners as novel drivers for functional remediation in contaminated soils

**DOI:** 10.3389/fpls.2015.00032

**Published:** 2015-02-05

**Authors:** Ying Teng, Xiaomi Wang, Lina Li, Zhengao Li, Yongming Luo

**Affiliations:** ^1^Key Laboratory of Soil Environment and Pollution Remediation, Institute of Soil Science, Chinese Academy of SciencesNanjing, China; ^2^Key Laboratory of Coastal Zone Environmental Processes, Yantai Institute of Coastal Zone Research, Chinese Academy of SciencesYantai, China

**Keywords:** rhizobia, nitrogen-fixation, bioremediation, organic pollutants, heavy metals, rhizospheric degraders, transgenic rhizobia

## Abstract

Environmental pollutants have received considerable attention due to their serious effects on human health. There are physical, chemical, and biological means to remediate pollution; among them, bioremediation has become increasingly popular. The nitrogen-fixing rhizobia are widely distributed in the soil and root ecosystems and can increase legume growth and production by supplying nitrogen, resulting in the reduced need for fertilizer applications. Rhizobia also possess the biochemical and ecological capacity to degrade organic pollutants and are resistant to heavy metals, making them useful for rehabilitating contaminated soils. Moreover, rhizobia stimulate the survival and action of other biodegrading bacteria, thereby lowering the concentration of pollutants. The synergistic action of multiple rhizobial strains enhances both plant growth and the availability of pollutants ranging from heavy metals to persistent organic pollutants. Because phytoremediation has some restrictions, the beneficial interaction between plants and rhizobia provides a promising option for remediation. This review describes recent advances in the exploitation of rhizobia for the rehabilitation of contaminated soil and the biochemical and molecular mechanisms involved, thereby promoting further development of this novel bioremediation strategy into a widely accepted technique.

## INTRODUCTION

Essential planetary functions such as primary production, the earth’s climate, biogeochemical and water cycling, and the maintenance of biodiversity have been severely undermined by anthropogenic activities (Teng et al., 2012; Alloway and Trevors, 2013; Valentín et al., 2013). Approximately 30% of the terrene environment is estimated to be degraded or contaminated, threatening agricultural production, and the environment (Alloway and Trevors, 2013; Valentín et al., 2013). In addition to contemporary pollutants such as heavy metals, hydrocarbons, and pesticides, a new generation of persistent organic pollutants (POPs) such as polybrominated diphenyl ethers (PBDEs), polychlorinated naphthalenes (PCNs), and perfluorooctanoic acid (PFOA) require urgent attention (Lohmann et al., 2007). Thus, there have been intensive studies investigating physico-chemical processes and bioaugmentation for their exploitation in multipurpose remediation technologies.

Although physico-chemical treatments (i.e., physical removal of contaminated soils, chemical extraction, and the application of chemical reagents) are still the most effective strategies to rapidly remediate heavily polluted sites, they are usually energy-intensive and intrusive for the environment (Segura and Ramos, 2013). In contrast, the less energy-demanding bioremediation utilizes living organisms and/or their bioproducts to clean up or stabilize inorganic/organic contaminants from the environment. Therefore, bioremediation is a promising alternative due to its relative low-level disturbance of contaminated sites, low cost and higher public acceptance compared with conventional remediation methods. Among the various types of bioremediation, phytoremediation is an environmentally friendly and cost effective approach that provides intangible benefits for the soil ecosystem, including soil carbon sequestration, soil quality improvement, biomass and biofuel production, and biodiversity maintenance (Rajkumar et al., 2012). Legumes are essential for nitrogen cycling in agriculture due to their symbiosis with the nitrogen-fixing rhizobia. Many reports have noted that some leguminous species are heavy-metal resistant and can significantly promote the dissipation of organic pollutants [i.e., polychlorinated biphenyls (PCBs), polycyclic aromatic hydrocarbons (PAHs), and amide herbicides; Fan et al., 2008; Hamdi et al., 2012; Carrasco-Gil et al., 2013; Li et al., 2013]. Intercropping of multiple leguminous plants may also become a promising *in situ* bioremediation strategy for contaminated sites (Sun et al., 2011a; Li et al., 2013).

The symbiosis between microorganisms and plants has been employed for the elimination of environmental contaminants to achieve high effectiveness and ecological sustainability. The effectiveness of phyto- or microbial-remediation is dependent on: (i) soil physio-chemical properties, such as pH, nutrient/organic matter content, soil surface properties, soil texture and bulk densities that influence plant–soil–water relationships and nutrient availability; (ii) toxicity or bioavailability of the targeted contaminants that reduce the productivity of the impacted soils, the biomass of plants and the degradative ability of microorganisms; (iii) plant species and traits; and (iv) the diversity and richness of the indigenous soil microbial communities or flora (Segura et al., 2009). However, these limitations can be addressed through the exploitation of the chemical interactions between the plants and the related rhizospheric microbes or endophytes (Abhilash et al., 2012). For the phytoremediation of heavy metals, heavy metal-resistant microbes can enhance plant growth, decrease metal phytotoxicity, and affect metal translocation and accumulation in plants (Li et al., 2012).

**FIGURE 1:**
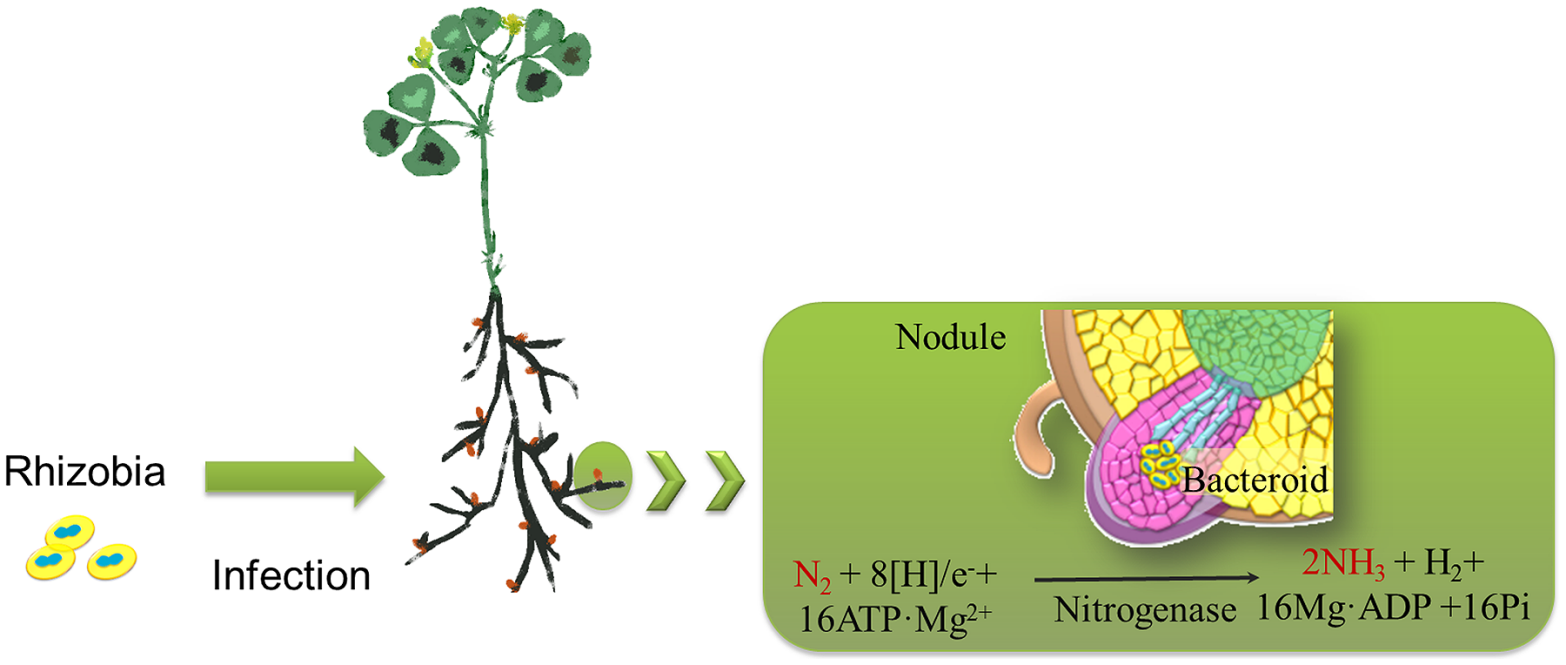
**Schematic drawing representing the nitrogen-fixing process associated with rhizobia.** Rhizobia invade the roots of legumes (i.e., alfalfa) and form nodules. During the process of biological nitrogen fixation in nodules, dinitrogen (N_2_) is reduced to two ammonia (NH_3_) molecules by the rhizobial nitrogenase. Hydrogen (H_2_) is a by-product of the symbiotic nitrogen fixation process.

*Rhizobiales,* belonging to the alphaproteobacteria, are Gram-negative bacteria of agronomic importance because some species form nitrogen-fixing symbiotic relationships with leguminous plants (Sato et al., 2005). Rhizobia invade the roots of legumes and form nodules to fix atmospheric nitrogen into ammonia, which is then provided to the host plants. This activity allows the plants to grow in the absence of an external nitrogen source (**Figure 1**; Prell and Poole, 2006; Deakin and Broughton, 2009). Hydrogen (H_2_) is a by-product of the symbiotic nitrogen fixation process and has recently been revealed to be a common element with novel bioactive properties that enhances plant tolerance to abiotic factors (i.e., oxidative stress and heavy metal toxicity; Cui et al., 2013; Jin et al., 2013). Because nitrogen is of the utmost importance for agricultural productivity, rhizobia have attained a special position in the field of agriculture as a plant growth promoter.

**FIGURE 2:**
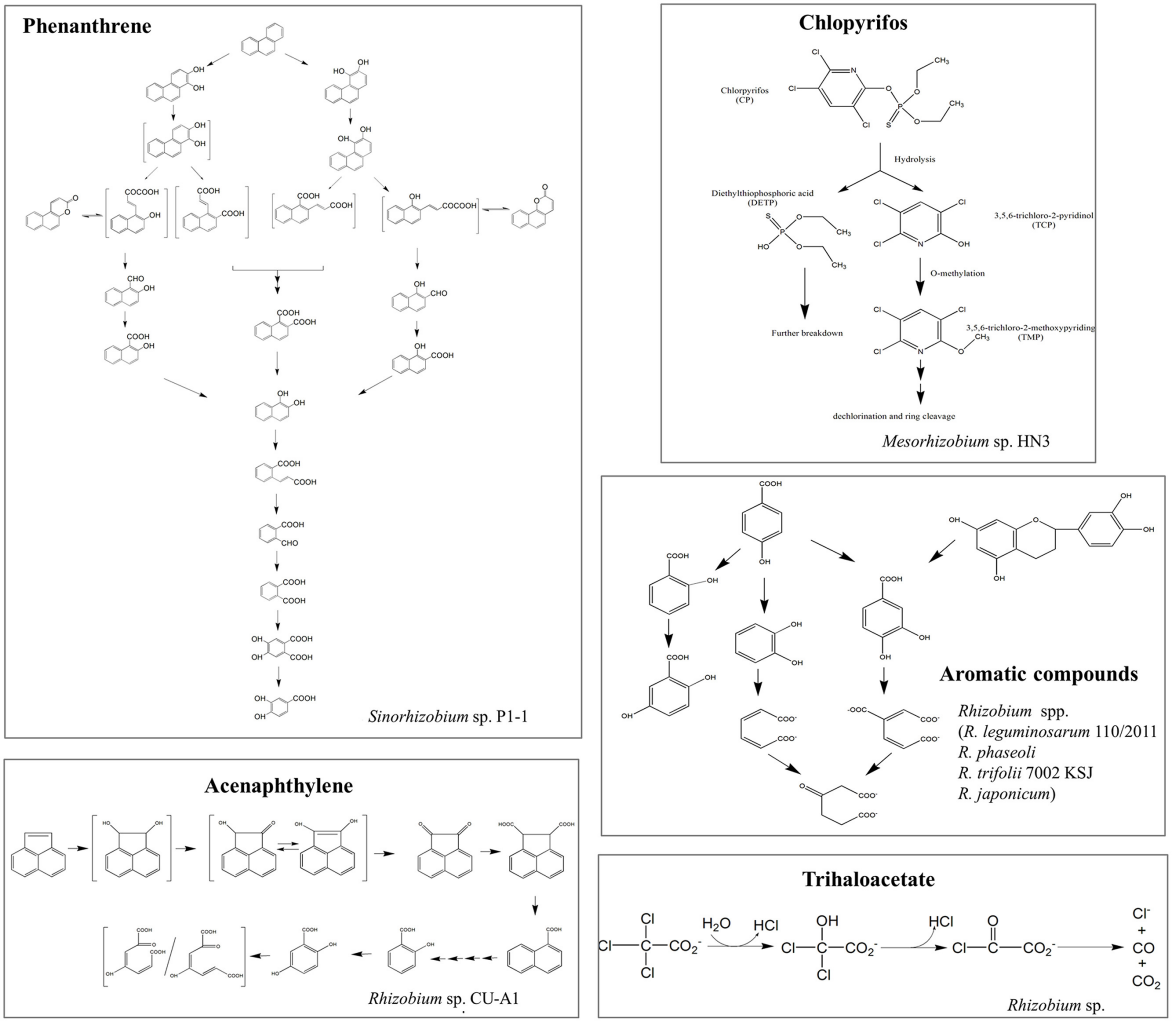
**Schematic representations of the proposed degradation pathways of several organic pollutants in rhizobial strains.** Organic pollutants: phenanthrene (Keum et al., 2006), chlorpyrifos (Jabeen et al., 2014), aromatic compounds (Muthukumar et al., 1982), acenaphthylene (Poonthrigpun et al., 2006), and trihaloacetate (Stringfellow et al., 1997). Structures in brackets represent the hypothetical metabolites.

Recently, rhizobia have been demonstrated to be available for the elimination of various types of organic pollutants from the environment, ranging from aromatic to linear hydrocarbons, chlorinated compounds, phenolic compounds, pesticides, and others (Table S1). Kaiya et al. (2012) reported that genus *Rhizobium* was one of the most abundant members of the degrading microcosm in dibenzofuran-contaminated soil. However, the bacterial catabolic enzymes and the pathways involved in the degradation of these compounds are only partially known (**Figure 2**). In addition to organic compounds, rhizobia have also been shown to have the potential to be a powerful tool for heavy metal bioremediation (Hao et al., 2014). Potential mechanisms involved are: (i) adsorption and accumulation of heavy metals; (ii) microbial secretion of enzymes and bioactive metabolites (i.e., extracellular polymeric substance, siderophores, and organic acids) to lessen their toxicity by altering the redox state of metals and increasing the complexation and bioavailability of metals; these actions can also indirectly aid phytoremediation (Hao et al., 2014); and (iii) microbial volatilization of heavy metals and their transformed products can also facilitate bioremediation, although this process has yet to be identified in rhizobia.

Due to copious production of plant biomass in terrestrial ecosystems, microbial symbionts constitute the ‘unseen majority’ during phytoremediation (Fester et al., 2014). The nitrogen-fixing and plant growth-promoting traits of rhizobia directly improve plant biomass, soil fertility, bioavailability of contaminants, the uptake, and translocation of pollutants from soil to plant, and the ability to degrade organic pollutants and indirectly help phytostabilize metals. These traits could help rhizobia overcome the constraints associated with phytoremediation (see Assisted-phytoremediation) and achieve higher efficiency (Hao et al., 2014); therefore, the symbiotic relationship between rhizobia and legumes results in an enhanced removal rate for pollutants (Glick, 2010). In comparison with rhizospheric microorganisms (including non-symbiotic diazotrophs), the balanced and stable endophytic association between rhizobia and their host plants provides a sustainable way to improve the performance of host plants in the context of the plant life cycle (Li et al., 2012). Thus, rhizobial remediation may represent a low-input biotechnology with no need for repeated inoculations of microbial agents. Some rhizospheric microbes capable of removing pollutants cannot survive and achieve bioremediation in the soil environment because they cannot compete with indigenous organisms. Bacterial inoculation agents also have advantages compared with fungal elicitors, such as a short period for culture and elicitation of responses in host plants (Wang et al., 2015). Moreover, beneficial bacteria often trigger relatively weaker defense responses than fungal elicitors, which might facilitate the sustainable and balanced relationships between the bioremediation partners. This might be attributed to the smaller niches that bacteria occupied than fungi (Wang et al., 2015). Another advantage of using rhizobia in organic pollutant bioremediation is that rhizobial nitrogen-fixation removes the limitation caused by nitrogen deficiency in sites. The deposition of hydrocarbon skeletons significantly increases the accumulation of organic carbon in the soil and generates a very high C/N imbalance during bioremediation (Moreto et al., 2005; Dashti et al., 2010). The presence of rhizobia can also exert direct or indirect impacts on microbial-degrader communities in the soil, thereby comprehensively facilitating restoration (Li et al., 2013). The mechanisms involved in this process include: (i) improvement of environmental conditions (i.e., pH) and nutrient availability (i.e., nitrogen) and (ii) changes in the amounts and constituents of root exudates due to the enhancement of plant metabolic activities following inoculation with rhizobia (Johnson et al., 2005; Terrence et al., 2011). Nevertheless, because hyperaccumulator plants are often limited in their plant-metabolic capacities with toxic contaminants, researchers have proposed that in most cases it is the plant-associated microorganisms that are the real players mediating the plants’ impacts on the completed transformation of contaminant (Fester et al., 2014).

In this review, we will introduce the latest findings in the field of bioremediation by rhizobia and summarize the knowledge of the restoration mechanism and modified strategies for rhizobia involved in ecosystem revitalization in contaminated sites.

## RHIZOBIA: WORKERS FOR BIOREMEDIATION

### DEGRADATION OF ORGANIC POLLUTANTS

Many free-living rhizobial strains in the genera *Agrobacterium*, *Rhizobium*, *Sinorhizobium*, and *Bradyrhizobium* have a demonstrated capacity to thrive in or utilize PAHs, PCBs, aromatic heterocycles (i.e., pyridine), or other toxic organic compounds (Keum et al., 2006; Poonthrigpun et al., 2006; Tu et al., 2011). Hussien et al. (1974) first isolated 22 strains of *Rhizobium* capable of degrading phenolic compounds (i.e., catechol, protocatechuic acid, *p*-hydroxybenzoic acid, and salicylic acid). Among them, *Rhizobium* sp. and *R. phaseoli* 405 dissimilated *p*-hydroxybenzoate to salicylate and then to gentisic acid before oxidation. Catechol and protocatechuic acid were also directly cleaved by these species, whereas *R. japonicum* converted catechin to protocatechuic acid (Muthukumar et al., 1982; Table S1).

Polycyclic aromatic hydrocarbons, a class of hazardous chemicals consisting of two or more fused benzene rings in various structural configurations, are listed as priority toxic pollutants by the U.S. Environmental Protection Agency due to their carcinogenicity, mutagenicity, and toxicity (Poonthrigpun et al., 2006). Ahmad et al. (1997) first isolated and characterized a variety of strains of *Rhizobium meliloti* in soils contaminated with aromatic/chloroaromatic hydrocarbons. They also found that the rhizobial population was composed of several phenotypically and genetically distinct strains and that all were effective in symbiotic N_2_-fixation. Acenaphthylene and phenanthrene are ubiquitous PAHs in the environment. Acenaphthylene (600 mg liter^-1^) can be totally degraded by *Rhizobium* sp. strain CU-A1 within three days through the naphthalene-1, 8-dicarboxylic acid metabolism pathway (Poonthrigpun et al., 2006). *Sinorhizobium* sp. C4 can utilize phenanthrene as a sole carbon source, and 16 intermediate metabolites involved in this degradation pathway have been identified (Keum et al., 2006).

Polychlorinated biphenyls are a class of POPs differing in the number of chlorine atoms (1–10) attached to their biphenyl rings (Passatore et al., 2014). Damaj and Ahmad (1996) revealed that rhizobia could act as a promising candidate for PCB degradation. Tu et al. (2011) demonstrated that *Sinorhizobium meliloti* ACCC17519 degraded more than 70% of 2,4,4′-TCB (PCB28), which was more efficient than the reported performance of other rhizobial strains. In experiments under aerobic conditions, 2-hydroxy-6-oxo-6-phenylhex-2,4-dienoic acid (HOPDA), the meta cleavage product in the classic PCBs-degradative pathway, was identified using GC-MS as the principal intermediate during the biotransformation of 2,4,4′-TCB by *S. meliloti*.

Some toxic aromatic acids and their hydroaromatic biosynthetic intermediates (i.e., quinate and shikimate) commonly distributed in plants and the rhizosphere have also been found to support the growth of rhizobia (Parke et al., 1985). Mimosine [β-*N*-(3-hydroxy-4-pyrid-one)-aminopropionic acid], an aromatic toxin produced by the roots of *Leucaena* sp., is toxic to both bacteria and eukaryotic cells (Awaya et al., 2005). Some *Leucaena*-nodulating *Rhizobium* strains have been reported to be able to utilize mimosine as a source of carbon and nitrogen (Soedarjo et al., 1995; Soedarjo and Borthakur, 1998), highlighting the catabolic capacity of aromatic compounds in rhizobia.

### DEGRADATIVE MECHANISMS INVOLVED IN ORGANIC POLLUTANTS

Because rhizobial strains are regularly exposed and adapted to recalcitrant pollutants, they are presumed to have developed enzymatic profiles essential for the degradation of these toxins (**Figure 2**; Zinjarde et al., 2014). Some aromatic acids and hydroaromatic biosynthetic intermediates (i.e., quinate and shikimate) commonly found within plants and in the rhizosphere could also support the growth of diverse rhizobial species (Parke et al., 1985). Indeed, genes for the catabolism of these compounds have been widely found in the genomes of rhizobiales and are conserved among different members. Linking rhizobial degradative gene expression to the decontamination and recycling of pollutants is crucial for illustrating the role played by these rhizobia in environment revitalization.

The microbial PCB-degradation system includes two major metabolic steps: (i) anaerobic reductive dechlorination, where PCBs are transformed into less chlorinated congeners; and (ii) aerobic breakdown of the biphenyl structure in lower-halogenated congeners (containing less than five chlorines), resulting in chloro-HOPDA (2-hydroxy-6-oxo-6-phenylhexa-2,4-dienoate), and the production of chlorobenzoic acid, ring opening, and potentially complete mineralization (Passatore et al., 2014). The oxidative pathways of PCB degradation in rhizobia have been documented in several studies. Aerobic rhizobial degradation of PCBs typically proceeds via the oxidative biphenyl pathway encoded by the *bph* genes that include a multi-component dioxygenase (*bph* A, E, F, and G), a dehydrogenase (*bph* B), a second dioxygenase (*bph* C), and a hydrolase (*bph* D) in other bacteria. Researchers first found that genomic DNAs from *Rhizobium* and *Bradyrhizobium* hybridized strongly with the *Comamonas testosteroni*-derived *bph*ABC gene probe, indicating the presence of a similar oxidative degradation system in rhizobia (Damaj and Ahmad, 1996; Ahmad et al., 1997). Xu et al. (2010) and Tu et al. (2011) showed that *R. meliloti* and *S. meliloti* could utilize 2,4,4′-TCB (PCB 28) as a sole carbon and energy source under aerobic conditions, and HOPDA has been identified as the main intermediate during the biotransformation of 2,4,4′-TCB by *S. meliloti*.

However, little is known about the dehalogenases responsible for the reductive dechlorination of PCBs in the aerobic rhizobia strains (Aken et al., 2010). Most dehalogenase-producing bacteria are anaerobic species and contain more than one dehalogenase. Leigh et al. (1988) reported that a fast-growing *Rhizobium* sp. utilized 2, 2-dichloropropionate (2, 2-DCP) and D, L-2-chloropropionate (DL-2CP) as sole sources of carbon and energy and contained three forms of inducible dehalogenases.

The degradation pathways of different rhizobial species vary. Muthukumar et al. (1982) reported that *p*-hydroxybenzoate was metabolized to protocatechuate that in turn was cleaved by protocatechuate 3, 4-dioxygenase via the ortho pathway in *R. leguminosarum*, *R. phaseoli*, and *R. trifolii*, whereas *R. japonicum* degraded *p*-hydroxybenzoate to catechol that was cleaved by catechol 1,2-dioxygenase. Moreover, the regulatory mechanisms for rhizobial bioremediation are related to various rhizobial catabolism pathways, thereby indicating that further investigations are needed. For example, the presence of glutamate favored the degradation of p-hydroxybenzoic and salicylic acids but had little effect on catechol during the rhizobial degradation of aromatic compounds (Hussien et al., 1974).

Researchers have also proposed that although some rhizobial species carry DNA sequences that are homologous to degradative genes, they may not be involved in the degradation process and may instead function in some other capacity (Ahmad et al., 1997). Sato et al. (2005) showed that *M. loti* MAFF303099 and *Bradyrhizobium japonicum* USDA110 possessed dehalogenase-like opening reading frame fragments (ORFs) in their genomes and produced functional haloalkane dehalogenases, but they did not function as halogenated compound degraders. Other researchers have reported that some strains of rhizobia (i.e., *S. meliloti*) did not possess genes for the bioremediation of pollutants (Chen et al., 2005). These outcomes could be due to the inefficient induction of degradative genes limited by plant exudates, O_2_-tension, cell density signals, and other environmental factors, the genetic background of the rhizobia, the interdependent regulation of the genes, the flux of metabolic intermediates, or the presence of their end products and non-specific transformation products (Damaj and Ahmad, 1996).

### REMEDIATION OF METALLIC CONTAMINANTS

Metals and metalloids are persistent toxins in organisms at high concentrations due to the fact that they are non-degradable and irreversibly immobilized in the environment. The selective pressure exerted by metals on microorganisms results in microbial populations with a high tolerance to metals as well as some tolerant metal hyper-accumulators (Pereira et al., 2006). There has been increasing concern over heavy metal resistance (i.e., arsenic, cadmium, zinc, copper, and lead) in free-living or symbiotic rhizobia and its effects on their potential for bioremediation (Hao et al., 2014). Heavy metal-resistant strains are commonly isolated from nodules of the metallicolous legume (i.e., *Robinia pseudoacacia*, *Anthyllis vulneraria,* and *Glycine max*) from mining tailings or contaminated sites. Smith and Giller (1992) first isolated strains of heavy metal-resistant *R. leguminosarum* from contaminated sewage sludge and mine spoils. Plant-growth promoting *Rhizobium* sp. and *Bradyrhizobium* sp. are the most common rhizobial strains identified at heavy metal-contaminated sites (Sriprang et al., 2002).

The mechanisms involved in the metal-resistance system have been studied at the nucleic acid and protein level in many rhizobial species. The results of Hao et al. (2012) revealed that the *Mesorhizobium amorphae* CCNWGS0123 genome carried multiple genes potentially involved in copper resistance, including *cus*AB (encoding a resistance-nodulation-cell division protein family-type copper eﬄux system), genes encoding P1B-type ATPases involved in heavy metal transition and translocation, and operons encoding copper resistance determinants. *S. meliloti* has been reported to detoxify arsenic through an aquaglyceroporin (AqpS) that has a physiological function in arsenic detoxification and resistance (ARS) due to the presence of the *ars* genes (Yang et al., 2005). By using the comparative RNAseq-based approach, Maynaud et al. (2013) identified genes that specifically responded to zinc and cadmium and demonstrated that several genes encoding metal eﬄux and sequestration systems were significantly up-regulated; thus, these genes could be considered to be involved in the most widely represented mechanisms of rhizobial metal tolerance. Pereira et al. (2006) reported that metals (zinc, lead, cadmium, chromium, and nickel) negatively influenced bacterial protein profiles, especially for polypeptide expression, whereas in tolerant *Rhizobium* strains these alterations mostly increased correspondingly.

**FIGURE 3:**
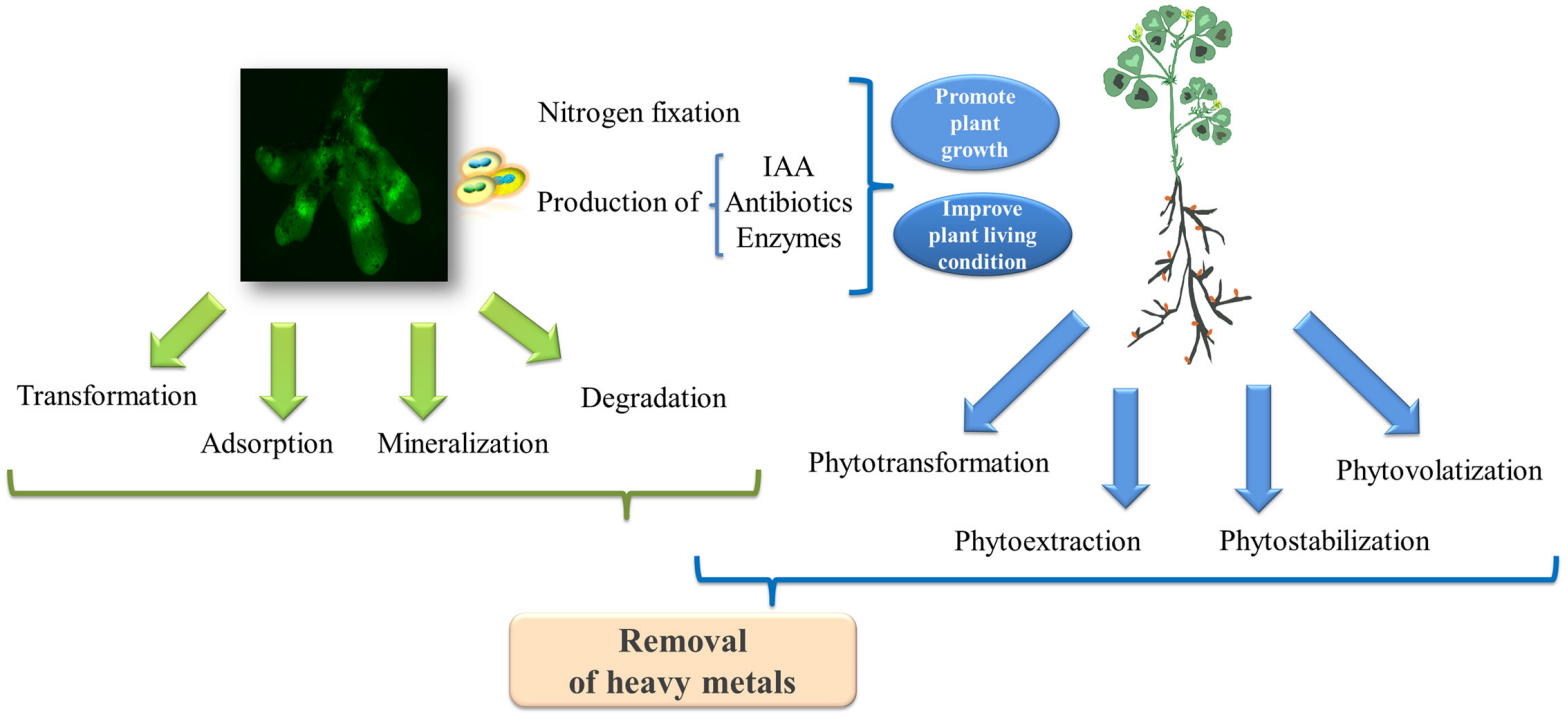
**The biodegradation mechanisms involved in the legume-rhizobia symbiosis for the removal of heavy metals.** The green fluorescent protein (GFP)-labeled rhizobia in the nodules was photographed by Chen Tu at the Yantai Institute of Coastal Zone Research, Chinese Academy of Sciences.

Based on studies in other bacteria, the metal resistance of rhizobia might be attributed to (**Figure 3**): (i) changes in the metal eﬄux of microbial cell membranes; (ii) intracellular chelation due to the production of metallothionein proteins (Nies, 1995); and (iii) the transformation of heavy metals to their less toxic oxidated forms through microbial metabolism (Nies, 2003). For example, the increased contents of reductive agents (i.e., glutathione concentrations) in microbial cells might reduce the toxicity of cadmium, thereby contributing to cadmium-resistance (Bright and Bulgheresi, 2010). Moreover, the metabolism of rhizobia also increases metal bioavailability in the soil through alterations in the soil pH, resulting in the release of chelators (i.e., siderophores) and organic acids capable of enhancing the complexation of metals and their mobility (Schalk et al., 2011). Microbial volatilization is another preferred method of metal bioremoval (i.e., selenium and mercury) in many rhizosphere bacteria (Souza et al., 2001; Zhang et al., 2012), although the mechanisms for the volatilization of metals in rhizobia have yet to be identified. Studies have suggested that engineering rhizobia for the volatilization of heavy metals could be a valuable avenue for tackling soil pollution. For example, Chen et al. (2014) demonstrated that *Pseudomonas putida* KT2440 endowed with the *arsM* gene encoding the As(III) *S*-adenosylmethionine (SAM) methyltransferase from *Rhodopseudomonas palustris* could remove arsenic from contaminated soil through microbial arsenic methylation and volatilization.

## ASSISTED-PHYTOREMEDIATION

Taking advantage of microbe-plant cross-talk offers a low-input biotechnology for ecosystem revitalization in toxic and nutrient-limited environments (bio-augmentation; Abhilash et al., 2012). Bacterial bioremediation in the field is often affected by a variety of factors, including microbial competition, fluctuating environmental conditions, and limited nutrients (Chen et al., 2005). Thus, the selection of effective contaminant-degrading bacteria alone is not enough to ensure optimal remediation. Moreover, phytoremediation is often regarded as slow and incomplete due to limitations in plant-metabolic capacities, rooting depths, and the seasonality of plant growth (Abhilash et al., 2012). It has also been proposed that the storage and accumulation of organic pollutants (or their metabolites) and toxic metals in plant tissues reduces plant survival and results in atmospheric contamination via volatilization through the leaves (Abhilash et al., 2012). Therefore, much work is still required for phytoremediation to achieve an effective performance within a reasonable time frame (Glick, 2010; Fester et al., 2014). The inclusion of microorganisms interacting with plants will address the weaknesses of the two individual systems. Legumes are considered to be pioneer plants for phytoremediation (Hao et al., 2014). The huge variety in the metabolic pathways employed by microbes makes them valuable tools to assist phytoremediation. The endophytic helper rhizobia, acting as ‘microbial logistics,’ break down contaminants that have accumulated in nodules, greatly reducing phytovolatilization, and facilitating phytoremediation in the rhizosphere and other environments. Sun et al. (2011b) showed that the accumulation of PCBs was higher in nodules than any other part of the alfalfa plant, indicating that high concentration of PCBs accumulated in alfalfa nodules. Furthermore, Li et al. (2013) demonstrated that *Rhizobium* was effective in removing PCBs when inoculated with alfalfa in pot experiments.

Legumes can enhance or stabilize rhizobial degradation and the biotransformation of various pollutants, which offers many advantages (de-Bashan et al., 2011): (i) the stimulation of legume growth conferred by the plant growth-promoting traits of rhizobia, including nitrogen fixation, phosphorus solubilization, phytohormone synthesis, siderophore release, and the production of ACC deaminase and volatile compounds (i.e., acetoin and 2, 3-butanediol); (ii) the phytostabilization and phytoextraction of heavy metals by the combined actions of the plants and microbes; (iii) the immobilization of contaminants and increasing soil organic content via root exudates; (iv) the provision of additional nitrogenous compounds to the soil, thereby improving soil fertility and supporting biological growth; and v) the modification of the structure and diversity of microflora [i.e., rhizobacteria and arbuscular mycorrhiza fungi (AMF)], which help immobilize metals, promote microbial degradation, and enhance the growth and phyto-stabilization of the legumes.

Various species of rhizobia are appropriate candidates for the phytoremediation of organic contaminants. The phytoremediation of organic contaminants mainly occurs by three mechanisms: phytoextraction, phytodegradation, and phytovolatilization. These three mechanisms demand a high biomass of plants and high availability of pollutants, both of which can be achieved by the N-fixation process and the secretions of rhizobia (Johnson et al., 2004). For example, the collaboration between *Medicago sativa* (alfalfa) and *S. meliloti* for the removal of aromatic pollutants has been extensively studied. Mehmannavaza et al. (2002) reported that a combined treatment (*S. meliloti* A-025 and alfalfa grown together) was the most effective for PCB biotransformation after 44 days. However, after 270 days alfalfa grown alone became the most effective treatment, whereas *S. meliloti* alone was the least effective. Xu et al. (2008) conducted further field experiments and found that PCB removal from rhizosphere soil was clearly enhanced in alfalfa simultaneously inoculated with *R. meliloti* at 90 days after planting. Similarly, in a pot study Teng et al. (2011) found that planting alfalfa inoculated with *R. meliloti* significantly lowered the initial soil PAH concentrations by 51.4% compared with unplanted control soil.

The phytoremediation of heavy metals might be impeded by their low bio-availability due to their insolubility and soil-bound properties (DalCorso et al., 2013). A collaborative system consisting of metal(loid) resistant rhizobia and legumes holds promising potential for the removal of metal and metalloid ions (e.g., the *Mimosa pudica*-*Cupriavidus taiwanensis* symbionts system; Chen et al., 2008). Hao et al. (2014) reviewed the phytoremediation of heavy and transition metals aided by legume-rhizobia symbiosis and concluded that rhizobial metabolism increased: (i) the uptake and translocation of metals from soils to plants due to their increased bioavailability; (ii) microbial extracellular polymeric substance production and enzyme activities that could immobilize and/or change the redox state of metals to lessen their toxicity to plants (Hao et al., 2014; e.g., *Rhizobium* sp. RP5 enhancement of Ni and Zn bioavailability to leguminous plants through the secretion of siderophores (Wani et al., 2007)); and (iii) the promotion of the leguminous biomass and bioaccumulation of metals in plants (Carrasco et al., 2005). A variety of metal(loid) resistance genes as well as genes reported to promote host plant growth were identified in a draft genome sequence, indicating their potential for aiding phytoremediation (Hao et al., 2012). For example, inoculation with *Bradyrhizobium* sp. ameliorated cadmium, zinc, and nickel stress in green gram (*Vigna radiata*) or annual ryegrass (*Lolium multiflorum*) by increasing the bioavailability of cadmium and the reduction of the metal concentrations in the plant organs and was accompanied by an increase in the plant biomass (Wani et al., 2007; Guo and Chi, 2014).

Mixed systems with multiple plant species or microbial symbionts have also been used for restoration, such as in PAH-spiked (chrysene-amended) agricultural soil (Johnson et al., 2004). Moreover, different legume species respond distinctly to the inoculation of metal-resistant rhizobia. Cadmium concentrations in the roots of *L. multiflorum* increased following inoculation with *Bradyrhizobium* sp. YL-6, but significantly decreased in the roots and shoots of *Glycine max* (Guo and Chi, 2014). Moreover, Camargo et al. (2011) found that inoculation with *Rhizobium* sp. produced differences in the growth of *Stizolobium deeringianum* Bort in atrazine-contaminated soil, whereas no differences were found in the growth of *S. aterrimum* Piper & Tracy.

## OTHER STRATEGIES TO ADDRESS RECALCITRANT POLLUTANTS

### OPTIMIZATION OF POLLUTANT-DEGRADING MICROBIAL CONSORTIA

The augmentation of the diversity and richness of degrading microbial consortia in contaminated sites has been regarded as one of the key reasons that rhizobia enhance the biodegradation of organic pollutants. By manipulating sterile and non-sterile soils, Johnson et al. (2004) found that the removal of chrysene was not caused by the direct degradation or uptake by *R. leguminosarum* bv. trifolii itself, but as a result of the stimulated plant growth and rhizospheric microflora promoted by the symbiotic association between white clover/ryegrass and this rhizobial strain. Many studies have suggested that the symbiotic association between alfalfa and rhizobia (i.e., *S. meliloti*) increases the counts of culturable PAH-degrading bacteria, soil microbial activity and the carbon utilization ability of the soil microbial community (Teng et al., 2011).

Rhizobia have the potential to directly modify rhizosphere microflora by improving environmental conditions and nutrient availability. Nitrogen is a major limiting factor in bioremediation and is often added to contaminated soils to stimulate the existing microbial communities (Terrence et al., 2011). Thus, the organic nitrogen resource fixed by rhizobia is one of the key factors that facilitates the growth and activity of other soil biodegraders. That being said, changes in the amounts and constituents of root exudates (i.e., organic acids, vitamins, and hormones) and secondary metabolites of legumes due to the colonization by rhizobia could be an additional promoting element for the optimization of rhizospheric microflora (Johnson et al., 2005).

### SYNERGISTIC INTERACTIONS WITH OTHER MICROBES

The collaboration between multiple beneficial microbes has been exploited for more comprehensive and sustainable rehabilitation. Simultaneous inoculation of multiple beneficial microbes often provides complementary and additive benefits to plants, revealing the compatibility, and synergy between distinct mutualists (Larimer et al., 2012). The synergistic promotion of plant biomass and activities of indigenous microbial species caused by dual colonization of rhizobia and other microbes could be a potent tool to further intensify bioremediation efficiency. For example, arbuscular mycorrhizal fungus (AMF) could form extended mycelial networks that not only provide organic nitrogen to host plants but also possess the catabolic capacity to remove organic pollutants (Harms et al., 2011). Teng et al. (2010) reported that the co-inoculation of *Rhizobium* sp. and AMF enhanced the removal rate of PCBs. This could be due to their combined contribution to plant growth and development by improving nutrient conditions (Saini et al., 2004) and the additive degradative ability of contaminants conferred by the two cleaners (Harms et al., 2011). The positive synergism of these interactions could be limited by plant species and soil conditions. Additionally, combined approaches using both biostimulation (with exogenous carbon sources) and bioaugmentation may be necessary to sustain the timely and effective *in situ* microbial biodegradation of pollutants (Andeer et al., 2013).

However, questions remain due to the considerable complexity of the soil environment. For example, the determination of the best method to establish appropriate numbers of foreign pollutant-degrading bacteria in the contaminated sites and to ensure that multiple degraders co-work during the entire rehabilitation process requires further studies (Segura and Ramos, 2013).

### TRANSGENIC RHIZOBIA IN BIOREMEDIATION

Recent advances in ‘omics’ technologies have provided opportunities to exploit genomic, transcriptomic, proteomic, and metabolomic means to modify the traits of ‘biological designers’ in order to maximize their phytoremediation efficiency (Abhilash et al., 2012). Bioengineering could potentially be used to manipulate the tolerance, accumulation, and degradation potentials of plants and microbes against pollutants.

Rhizobial transgenics can be harnessed for the accumulation of inorganic contaminants and detoxification of organic pollutants. Ike et al. (2007) transferred two resistance genes [synthetic tetrameric metallothionein (*MTL4*), and a cDNA encoding the phytochelatin synthase from *Arabidopsis thaliana* (*AtPCS*)] into *M. huakuii* subsp. rengei B3. The results from this study showed that the two recombinant strains accumulated more cadmium compared with the free-living cells. When *Astragalus sinicus* was inoculated with the two recombinant strains, the increased cadmium accumulation in nodules was observed. However, the accumulation of copper was not promoted by the expression of *MTL4* in *M. huakuii* B3 in nodules of *A. sinicus*, indicating that the functioning of the *MTL4* gene might vary in response to different metal stresses (Sriprang et al., 2002). Genetic horizontal transfer of plasmid pJP4 that encodes genes for mercury resistance and 2,4-D degradation into *Bradyrhizobium* in non-sterile soil (Kinkle et al., 1993) reportedly resulted in the co-metabolism of herbicide 2,4-D (Feng et al., 1994) and the enhancement of microbial PCB degradation in the soil (Donelly et al., 1994). The work of Chen et al. (2005) showed that when corresponding degradative genes were introduced into *S. meliloti*, the degradation rate of PCBs increased in the transformed strain. The dechlorination of PCBs by alfalfa inoculated with the *S. meliloti* transformants was more than twice that of alfalfa grown with wild-type *S. meliloti*. Therefore, the effectiveness of phytostabilization could be improved by developing such downstream functional strategies.

Studies have shown that the simultaneous expression of multiple genes related to degradation or metal accumulation exerts additive effects on the removal of pollutants (Ike et al., 2007). However, it is difficult to control the expression levels of the transferred genes in the recipient cells and the effect of certain individual genes may be limited to a narrow scope of impact as a consequence, thereby restricting its application. Further exploitation of the genomic databases of these rhizobia and the identification of the various functions of different genes are required in order to identify the most effective genes for bioremediation.

## CONCLUSION

Rhizobia have been recognized as a potential strategy to simultaneously enhance soil nitrogen content, reduce the use of fertilizers, and increase H_2_ concentration (hydrogen fertilizers) in the rhizosphere through symbiotic nitrogen fixation. Rhizobia also possess the biochemical and ecological capacity to degrade environmental organic chemicals and to decrease the risk associated with metals and metalloids in contaminated sites. Rhizobia-assisted phytoremediation provides further environmental and economic benefits for bioremediation. The exploitation of microbe–microbe or plant–microbe interactions between intra-species and inter-species communication in the rhizosphere could represent more integrative approaches to further facilitate bioremediation. Researchers have proposed that the wide adoption of these biological adaptation strategies would result in the development of environmentally friendly management techniques (i.e., biological carbon sequestration, bioenergy, and bioremediation: the “3B” technique) to further enhance biodiversity and relieve environmental stressors (Teng et al., 2012).

Symbiotic nitrogen fixation and the reductive dechlorination of organic pollutants are both oxygen-sensitive and energetically costly for rhizobia (Morris and Schmidt, 2013). The presence of leghemoglobin maintains the low O_2_ concentrations in the root nodules (within the nanomolar range) and protects nitrogenase from inhibition by O_2_ (Becker et al., 2004). Therefore, researchers have proposed that the micro-oxic environment formed in nodules might also provide the proper conditions for the reductive degradation of organic pollutants. Moreover, *Rhizobium* sp. have a demonstrated capacity for partial denitrification in soils; some strains have been associated with nitrate reductase (NAR) activities, especially under micro-oxic conditions (Streeter and DeVine, 1983). Some reports have provided evidence of NAR-mediated dechlorination of PCB153 in free-living cells of *Phanerochaete chrysosporium* and crude enzyme extracts from *Medicago sativa* leaves, even under aerobic conditions (De et al., 2006; Magee et al., 2008). O’Hara et al. (1983) reported that *S. meliloti* possessed denitrifying activities in both its free-living and symbiotic forms. However, Becker et al. (2004) only found two genes (*nir*V and *nor*B) that were induced in the *S. meliloti* bacteroids (Becker et al., 2004). Therefore, the involvement of metabolic enzymes in the potential degradation of organic compounds in rhizobia requires further study.

Our understanding of the genetic and molecular influences of bioremediation effects is not complete, and the goal of transforming this strategy into practice has not yet been fully achieved. The suitable selection of rhizobial strains or consortia in combination with plant hosts, indicators of successful bioremediation under field conditions and the mechanisms involved constitute future work that should be pursued for the initiation of successful efforts in this area. The successful execution of this versatile bioremediation strategy also requires a thorough understanding of the factors regulating the growth, metabolism, and functions of degradative rhizobia and indigenous microbial communities at contaminated sites. Furthermore, to date the major work of the “Rhizobial bioremediation” field has been mostly conducted under controlled laboratory conditions and not in the field, where further practical investigations and testing are required before bioremediation can become a widely accepted technique. The selection of suitable rhizobial strains will be necessary for the remediation of certain polluted sites.

In conclusion, this review provides a comprehensive framework for applying the versatile rhizobia to revitalize contaminated soils. The selective introduction of degradative rhizobia into hyperaccumulator plants could facilitate the accelerated removal of mixed pollutants from soils. Using this approach, the exploitation of these degradative, nitrogen-fixing and endophytic pollutant-cleaners could become a highly efficient, eco-friendly and low-input bioremediation technology for the future.

## Conflict of Interest Statement

The authors declare that the research was conducted in the absence of any commercial or financial relationships that could be construed as a potential conflict of interest.
