# Supplementary material for: Rhizobia and their bio-partners as novel drivers for functional remediation in contaminated soils
Source: Front Plant Sci. 2015 Feb 5;6:32. doi: 10.3389/fpls.2015.00032 (PMC4318275; doi:10.3389/fpls.2015.00032)
Supplement: Supplementary file 1 [file Table_1.DOCX]

**Table S1** Overview of the natural and genetically modiﬁed rhizobia studied for the removal of contaminants (organic pollutants and heavy metals)

| **Features / Pollutant** | **Origin** | **Rhizobia strains** | **Reference** | **Putative Mechanisms** | | |
| --- | --- | --- | --- | --- | --- | --- |
|  |  |  |  | Plant growth promotion | Rhizobial degradative/resistant ability | Stimulated  rhizospheric microflora |
| **Polycyclic aromatic hydrocarbons (PAHs)** | | | | | | |
| Chrysene | a PAH contaminated industrial soil | *Rhizobium leguminosarum* bv. trifolii | (Johnson et al., 2004) | √ | × | √ |
| Phenanthrene | a polycyclic aromatic hydrocarbon (PAH)-contaminated sites | *Sinorhizobium* sp. C4 | (Keum et al., 2006; Keum et al., 2008) | - | √ | - |
| Dibenzothiophene | - | *Rhizobium meliloti* | (Frassinetti et al., 1998) | - | √ | - |
| Chlorophenol | pentachlorophenol (PCP)-contaminated soils | *Rhizobium* sp. 4-CP-20 | (Yang and Lee, 2008) | - | √ | - |
| PAHs | - | *Rhizobium meliloti* (strain ACCC 17519) | (Teng et al., 2011) | √ | √ | √ |
| **Halo compounds** | | | | | | |
| Polychlorinated biphenyls (PCBs) | - | *Rhizobium meliloti* Zb57 (1996), *Sinorhizobium meliloti* A-025 (2002) | (Damaj and Ahmad, 1996; Mehmannavaza et al., 2002) | - | √ | - |
| PCBs (PCB-Congener-Mix) | noduels of *Medicago sativa* | *Rhizobium meliloti*  (strain ACCC 17519) | (Xu et al., 2010) | √ | - | √ |
| 2,4,4`-TCB | noduels of *Medicago sativa* | *Sinorhizobium meliloti* (ACCC17519) | (Tu et al., 2011) | - | √ | √ |
| PAH PCB | - | *Rhizobium meliloti* | (Ahmad et al., 1997) | - | √ | - |
| Trihaloacetate | - | *Rhizobium* sp. | (Stringfellow et al., 1997) | - | √  (Haloalkanoate dehalogenase I1, DehE) | - |
| Haloalkanoic acid | - | *Rhizobium* sp. RC1 | (Hamid et al., 2013) | - | √  (Haloalkanoic acid dehalogenase) | - |
| Haloalkane | - | *Mesorhizobium loti* MAFF303099, *Bradyrhizobium japonicum* USDA110 | (Sato et al., 2005) | - | √  (Haloalkane dehalogenases) | - |
| **Phenolic compounds** | | | | | | |
| Catechol， protocatechuic acid | - | *Rhizobium phaseoli* 405 | (Hussien et al., 1974) | - | √ | - |
| Phenanthrene, benzo[a]pyrene | *Phaseolus vulgaris* | *Rhizobium tropici* CIAT 899 | ^(^Yessica et al., 2013^)^ | - | √ | - |
| Haloacetic acids (chloroacetic acid and triﬂuoroacetic acid) | - | *Sinorhizobium meliloti* 1021 | (Sallabhan et al., 2013) | - | √ (Haloacid dehalogenase) | - |
| Benzophenone, 4-hydroxybenzoic acid, phenol | *Astragalus chrysopteru* | *Rhizobium sp.*  CCNWTB 701 | (Wei et al., 2008) | - | √ | - |
| **Heterocyclic aromatic compounds** | | | | | | |
| Pyridine |  | *Rhizobium* sp. NJUST18 | (Shen et al., 2014; Poonthrigpun et al., 2006) | - | √ | - |
| Acenaphthylene | Petroleum-contaminated soil | *Rhizobium* sp. strain CU-A1 |  | - | √ | - |
| **Pesticides** | | | | | | |
| Vitavax, Rizolex | - | *Bradyrhizobium* sp. isolate 8, *Rhizobium leguminosarum* biovar trifolii strain TA1 | (Moawad et al., 2014) | - | √ | - |
| Atrazine | - | *Rhizobium* sp. PATR | (Bouquard et al., 1997) | - | √ | - |
| Chlorpyrifos, 3, 5, 6-trichloro-2-pyridinol | - | *Mesorhizobium* sp. HN3 | (Jabeen et al., 2014) | - | √ | - |
| Dalapon (2,2-Dichloropropionic Acid) |  | *Rhizobium* sp. | (Huyop and Cooper, 2012) | - | √ | - |
| **Other organic toxins** | | | | | | |
| 2,3-Dichloro-1-propanol | - | *Rhizobium*-*Agrobacterium* strains, NHC2 and NHG3, | (Effendi et al., 2000) | - | √ | - |
| Poly-3-hydroxybutyrate | - | *Rhizobium* (*Sinorhizobium*) *meliloti* | (Aneja and Charles, 1999) | - | √ | - |
| 3-Hydroxy-4-pyridone | - | *Rhizobium* sp. strain TAL1145 | (Awaya et al., 2005) | - | - | - |
| **Heavy metals-Resistance** | | | | | | |
| Arsenic | *Medicago sativa* | *Sinorhizobium meliloti* | (Hao et al., 2014) | - | - | - |
| Arsenic | *Medicago sativa* | *Sinorhizobium meliloti* | (Yang et al., 2005) | - | - | - |
| Cadmium | *Lolium multiflorum* Lam.  and *Glycine max* (L.) Merr. | *Bradyrhizobium* sp. YL-6 | (Guo et al., 2014) | - | - | - |
| Copper | *Mimosa pudica* | *Cupriavidus taiwanensis* TJ208 | (Chen et al., 2008) | - | √ | - |
| Copper | *Medicago lupulina* in mine tailings | *Sinorhizobium meliloti* CCNWSX0020 | (Fan et al., 2011) | - | √ | - |
| **Genetically modified strains** | | | | | | |
| 2`,3,4-PCB , 2,4-dinitrotoluene | *Medicago sativa* | *Sinorhizobium meliloti* transferred with ortho- halobenzoate 1,2-dioxygenase (ohb) genes | (Chen et al., 2005) | - | √ | √ |
| Cadmium resistance | *Astragalus sinicus* | transferred *Mesorhizobium* huakuii subsp. rengei B3 | (Ike et al., 2007) | - | - | - |
| Copper resistance | *Medicago lupulina* | *Transferred Sinorhizobium meliloti* CCNWSX0020 | (Li et al., 2014) | - | - | - |

‘-‘ Means unknown; ‘√’ indicates the strain has the ability; ‘×’ indicates the strain does not have the ability.

**References**

Ahmad, D., Mehmannavaz, R., and Damaj, M. (1997). Isolation and characterization of symbiotic N_2_-fixing *Rhizobium meliloti* from soils contaminated with aromatic and chloroaromatic hydrocarbons: PAHs and PCBs. *Int. Biodeter. Biodegr.* 39, 33-43. doi: 10.1016/S0964-8305(96)00065-0

Aneja, P. and Charles, T. C. (1999). Poly-3-hydroxybutyrate degradation in *Rhizobium* (*Sinorhizobium*) *meliloti*: isolation and characterization of a gene encoding 3-hydroxybutyrate dehydrogenase. J. Bacteriol. 181, 849-857. doi: 0021-9193/99/$04.0010

Awaya, J. D., Fox, P. M., and Borthakur, D. (2005). pyd Genes of *Rhizobium* sp. strain TAL1145 are required for degradation of 3-hydroxy-4-pyridone, an aromatic intermediate in mimosine metabolism. *J. Bacteriol.* 187, 4480-4487. doi: 10.1128/JB.187.13.4480-4487.2005

Bouquard, C., Ouazzani, J., Prome, J., Michel-Briand, Y., and Plesiat, P. (1997). Dechlorination of atrazine by a *Rhizobium* sp. isolate. *Appl. Environ. Microbiol.* 63: 862-866. doi:10.4236/as.2014.57065

Chen, W. M.，Wu, C. H.，James, E. K.，and Chang, J. S. (2008). Biosorption capability of *Cupriavidus taiwanensis* and its effects on heavy metal removal by nodulated *Mimosa pudica*. *J. Hazard. Mater.* 151, 364-371. doi:10.1016/j.jhazmat.2007.05.082

Chen, Y. Q., Adam, A., Toure, O., and Dutta, S. K. (2005). Molecular evidence of genetic modiﬁcation of *Sinorhizobium meliloti*: enhanced PCB bioremediation. *J. Ind. Microbiol. Biotechnol.* 32, 561-566. doi: 10.1007/s10295-005-0039-2

Damaj, M., and Ahmad, D. (1996). Biodegradation of polychlorinated biphenyls by rhizobia: a novel finding. *Biochem. Bioph. Res. Co.* 218, 908-915. doi: 10.1006/bbrc.1996.0161

Effendi, A. J., Greenaway, S. D. and Dancer, B. N. (2000). Isolation and characterization of 2,3-dichloro-1-propanol-degrading rhizobia. Appl. Environ. Microbiol. 66, 2882-2887. doi: 10.1016/j.ijmm.2006.02.008

Fan, L., M., Ma, Z. Q., Liang, J. Q., Li, H. F., Wang, E. T., and Wei, G. H. (2011). Characterization of a copper resistant *Sinorhizobium meliloti* CCNWSX0020 isolated from *Medicago lupulina* in mine tailings. *Bioresource Technol.* 102, 703-709. doi:10.1016/j.biortech.2010.08.046

Frassinetti, S., Setti, L., Corti, A., Farrinelli, P., Montevecchi, P., and Vallini, G. (1998). Biodegradation of dibenzothiophene by a modulating isolate of *Rhizobium meliloti*. *Can. J. Microbiol.* 44, 289-297. doi: 10.1139/w97-155

Guo, J. K., and Chi, J. (2014). Effect of Cd-tolerant plant growth-promoting rhizobium on plant growth and Cd uptake by *Lolium multiflorum* Lam. and *Glycine max* (L.) Merr. in Cd-contaminated soil. *Plant Soil* 375, 205-214. doi: 10.1007/s11104-013-1952-1

Hamid, A. A. A., Hamid, T. H. T. A., Wahab, R. A., and Huyop, F. (2013). Identiﬁcation of functional residues essential for dehalogenation by the non-stereospeciﬁc a-haloalkanoic acid dehalogenase from *Rhizobium* sp. RC1. *J. Basic Microbiol.* 53, 1-7. doi: 10.1002/jobm.201300526

Hao, X., Taghavi, S., Xie, P., Orbach, M. J., Alwathnani, H. A., Rensing, C., et al. (2014). Phytoremediation of heavy and transition metals aided by legume-rhizobia symbiosis. *Int. J. Phytoremediat.* 16, 179-202. doi: 10.1080/15226514.2013.773273

Hussien, Y. A., Tewfik, M. S., and Hamdi, Y. A. (1974). Degradation of certain aromatic compounds by rhizobia. *Soil Biol. Biochem.* 6, 377-381. doi: 10.1016/0038-0717(74)90047-9

Huyop, F. and Cooper, R. (2012). Degradation of millimolar concentration of the herbicide dalapon (2,2-dichloropropionic acid) by *Rhizobium* sp. isolated from soil, *Biotechnol. Biotec. Eq.* 26, 3106-3112. doi: 10.5504/BBEQ.2012.0058

Ike, A., Sriprang, R., Ono, H., Murooka, Y., and Yamashita, M. (2007). Bioremediation of cadmium contaminated soil using symbiosis between leguminous plant and recombinant rhizobia with the MTL4 and the PCS genes. *Chemosphere* 66, 1670-1676. doi: 10.1016/j.*Chemosphere*.2006.07.058

Jabeen, H., Iqbal, S. and Anwar, S. (2014). Biodegradation of chlorpyrifos and 3, 5, 6-trichloro-2-pyridinol by a novel rhizobial strain *Mesorhizobium* sp. HN3. *Water Environ. J.* doi:10.1111/wej.12081

Johnson, D. L., Maguire, K. L., Anderson, D. R., and McGrath, S. P. (2004). Enhanced dissipation of chrysene in planted soil: the impact of a rhizobial inoculum. *Soil Biol. Biochem.* 36, 33-38. doi: 10.1016/j.soilbio.2003.07.004

Keum, Y. S., Seo, J. S., Hu, Y. T., and Li, Q. X. (2006). Degradation pathways of phenanthrene by *Sinorhizobium* sp. C4. *Appl. Microbiol. Biotechnol.* 71, 935-941. doi: 10.1007/s00253-005-0219-z

Keum, Y. S., Seo, J. S., Li, Q. X., and Kim, J. H. (2008). Comparative metabolomic analysis of *Sinorhizobium* sp. C4 during the degradation of phenanthrene. *Appl. Microbiol. Biotechnol.* 80, 863-872. doi: 10.1007/s00253-008-1581-4

Li, Z. F., Ma, Z. Q., Hao, X. L., Rensing, C., and Wei, G. H. (2014). Genes conferring copper resistance in *Sinorhizobium meliloti* CCNWSX0020 also promote the growth of *Medicago lupulina* in copper-contaminated soil. *Appl. Environ. Microbiol.* 80, 1961-1971. doi:10.1128/AEM.03381-13

Mehmannavaza, R., Prasher, S. O., and Ahmad, D. (2002). Rhizospheric effects of alfalfa on biotransformation of polychlorinated biphenyls in a contaminated soil augmented with *Sinorhizobium meliloti*. *Process Biochem.* 37: 955-963. doi: 10.1016/S0032-9592(01)00305-3

Moawad, H., El-Rahim, W.M., Shawky, H., Higazy, A.M. and Daw, Z.Y. (2014). Evidence of fungicides degradation by *Rhizobia*. *Agricultural Sciences*, 5, 618-624. http://dx.doi.org/10.4236/as.2014.57065

Poonthrigpun, S., Pattaragulwanit, K., Paengthai, S., Kriangkripipat, T., Juntongjin, K., Thaniyavarn, S., et al. (2006). Novel intermediates of acenaphthylene degradation by *Rhizobium* sp. strain CU-A1: evidence for naphthalene-1,8-dicarboxylic acid metabolism. *Appl. Environ. Microbiol.* 72, 6034-6039. doi: 10.1128/AEM.00897-06

Sallabhan, R., Kerdwong, J., Dubbs, J. M., Somsongkul, K., Whangsuk ,W., Piewtongon, P., et al. (2013). The *hdh*A gene encodes a haloacid dehalogenase that is regulated by the LysR-type regulator, HdhR, in *Sinorhizobium meliloti*. *Mol. Biotechnol.* 54:148-157. doi: 10.1007/s12033-012-9556-1

Sato, Y., Monincová, M., Chaloupková, R., Prokop, Z., Ohtsubo, Y., Minamisawa, K., et al. (2005). Two rhizobial strains, *Mesorhizobium loti* MAFF303099 and *Bradyrhizobium japonicum* USDA110, encode haloalkane dehalogenases with novel structures and substrate speciﬁcities. *Appl. Environ. Microb.* 71, 4372-4379. doi: 10.1128/AEM.71.8.4372-4379.2005

Shen, J. Y., Zhang, X., Chen, D., Liu, X. D., and Wang, L. J. (2014). Characteristics of pyridine biodegradation by a novel bacterial strain, *Rhizobium* sp. NJUST18. *Desalin. Water Treat.* DOI: 10.1080/19443994.2014.915585

Stringfellow, J. M., Cairns, S. S., Cornish, A., and Cooper, R. A. (1997). Haloalkanoate dehalogenase I1 (DehE) of a *Rhizobium* sp. molecular analysis of the gene and formation of carbon monoxide from trihaloacetate by the enzyme. *Eur. J. Biochem.* 250, 789-793. doi: 10.1111/j.1432-1033.1997.00789.x

Teng, Y., Shen, Y. Y., Luo, Y. M., Sun, X. H., Sun, M. M., Fu, D. Q. et al. (2011). Inﬂuence of *Rhizobium meliloti* on phytoremediation of polycyclic aromatic hydrocarbons by alfalfa in an aged contaminated soil. *J. Hazard. Mater.* 186: 1271-1276. doi: 10.1016/j.jhazmat.2010.11.126

Tu, C., Teng, Y., Luo, Y. M., Li, X. H., Sun, X. H., Li, Z. G., et al. (2011) Potential for biodegradation of polychlorinated biphenyls (PCBs) by *Sinorhizobium meliloti*. *J. Hazard. Mater.* 186, 1438-1444. doi: 10.1016/j.jhazmat.2010.12.008

Wei, G. H., Yu, J. F., Zhu, Y, H., Chen, Y. M., and Wang, L. (2008). Characterization of phenol degradation by *Rhizobium* sp. CCNWTB701 isolated from *Astragalus chrysopteru* in mining tailing region. *J. Hazard Mater.* 151, 111-117. doi: 10.1016/j.jhazmat.2007.05.058

Xu, L., Teng, T., Luo, Y. M., and Li, Z. G. (2010). Effects of *Rhizobium meliloti* on PCBs degradation and transformation in solution culture. *Environ. Sci.* 31, 255-259 (in Chinese).

Yang, C. F., and Lee, C. M. (2008). Enrichment, isolation, and characterization of 4-chlorophenol-degrading bacterium *Rhizobium* sp. 4-CP-20. *Biodegradation* 19, 329-336. doi: 10.1007/s10532-007-9139-1

Yang, H. C., Cheng, J. J, Finan, T. M., Rosen, B. P., and Bhattacharjee, H. (2005). Novel pathway for arsenic detoxiﬁcation in the legume symbiont *Sinorhizobium meliloti*. *J. Bacteriol.* 187, 6991-6997. doi: 10.1128/JB.187.20.6991-6997.2005

Yessica, G. P., Alejandro, A., Ronald, F-C., José, A. J., Esperanza, M-R., Samuel, C-S. J., et al. (2013). Tolerance, growth and degradation of phenanthrene and benzo[a]pyrene by *Rhizobium tropici* CIAT 899 in liquid culture medium. *Appl. Soil Ecol.* 63, 105-111. doi: 10.1016/j.apsoil.2012.09.010
